# Unattractive faces are more attractive when the bottom-half is masked, an effect that reverses when the top-half is concealed

**DOI:** 10.1186/s41235-022-00359-9

**Published:** 2022-01-24

**Authors:** Farid Pazhoohi, Alan Kingstone

**Affiliations:** Department of Psychology, University of British Columbia, 2136 West Mall, Vancouver, BC V6T 1Z4 Canada

**Keywords:** Facial attractiveness, Face mask, Full and half human faces, Age, Positivity bias, Upper- and lower-face

## Abstract

Facial attractiveness in humans signals an individual’s genetic condition, underlying physiology and health status, serving as a cue to one’s mate value. The practice of wearing face masks for prevention of transmission of airborne infections may disrupt one’s ability to evaluate facial attractiveness, and with it, cues to an individual's health and genetic condition. The current research investigated the effect of face masks on the perception of face attractiveness. Across four studies, we tested if below- and above-average attractive full faces are equally affected by wearing facial masks. The results reveal that for young faces (Study 1) and old faces (Study 2) a facial mask increases the perceived attractiveness of relatively unattractive faces, but there is no effect of wearing a face mask for highly attractive faces. Study 3 shows that the same pattern of ratings emerged when the bottom-half of the faces are cropped rather than masked, indicating that the effect is not mask-specific. Our final Study 4, in which information from only the lower half of the faces was made available, showed that contrary to our previous findings, highly attractive half-faces are perceived to be less attractive than their full-face counterpart; but there is no such effect for the less attractive faces. This demonstrates the importance of the eye-region in the perception of attractiveness, especially for highly attractive faces. Collectively these findings suggest that a positivity-bias enhances the perception of unattractive faces when only the upper face is visible, a finding that may not extend to attractive faces because of the perceptual weight placed on their eye-region.

**Supplementary Information:**

The online version contains supplementary material available at 10.1186/s41235-022-00359-9.

## Significance statement

Wearing face masks are generally used in healthcare settings by health professionals to prevent transmission of diseases. However, currently wearing of face-masks has extended globally to the general population to reduce the transmission of COVID-19. The practice of face masks on a daily basis can impair one's ability to perceive faces, and may affect perceptions of facial attractiveness. In the current research we investigated the effect of facial masks on the perceptions of facial attractiveness. Our results show that wearing face masks increase the perceived attractiveness of less attractive faces in both young and old people, while face masks do not affect highly attractive faces.

## Introduction

Facial attractiveness in humans is an honest signal of an individual’s genetic condition, underlying physiology and health status, and thus serves as a cue of one’s value as a potential mate (Thornhill & Gangestad, 1999). Importantly, humans have evolved psychological mechanisms to distinguish and discriminate facial features about such qualities (Little et al., 2011; Thornhill & Gangestad, 1999). For instance, men and women prefer symmetrical faces in the opposite sex as it also is able to signal genetic quality and healthy development (Grammer & Thornhill, 1994; Rhodes, 2006; Thornhill & Gangestad, 1993). Similarly, people prefer average faces, possibly because they signal genetic heterozygosity and resistance to disease (Grammer & Thornhill, 1994; Rhodes et al., 2001). Men and women also prefer sexually dimorphic faces in the opposite sex (i.e., feminine traits in female faces and masculine traits in male faces), as such features may be viewed as denoting genetic condition, hormonal profile, health and immunity (Little et al., 2011; Rhodes, 2006; Thornhill & Gangestad, 1999, 2006). Faces can also convey other information regarding one’s health, genetic condition and mate value, including skin health and color, adiposity and weight (De Jager et al., 2018; Little et al., 2011; Stephen et al., 2011).

Humans, as a social animal, may adhere to practices that cover some parts of the face. For example, some Muslim women may cover their faces by wearing face covering veils such as the niqab, which impairs facial expression recognition by observers (Fischer et al., 2012; Kret & De Gelder, 2012); it may also serve the intended purpose of decreasing women’s attractiveness by their male relatives (Pazhoohi, 2016; Pazhoohi & Kingstone, 2020). Another practice is wearing surgical masks (or simply face masks) in healthcare settings by health professionals to prevent transmission of airborne infections, and is presently practiced globally by the general population to reduce the transmission of COVID-19. Yet face masks also impair one's ability to perceive faces and the emotions that they convey (Carbon, 2020; Freud et al., 2020; Pazhoohi et al., 2021). It thus follows that the facial covering practices may impair one's ability to perceive signals of health and genetic condition, and hence, the attractiveness of others.

To the best of our knowledge, save for a study by Miyazaki and Kawahara (2016), no research has examined the effect of facial masks on the perception of attractiveness prior to COVID-19 pandemic. Miyazaki and Kawahara used a facial mask, as well as a notebook or a card, to occlude the lower part of faces and found that the facial mask reduced perceived attractiveness, while the other occluding objects increased it, suggesting that both factors can impact judgements of attractiveness. A facial mask may prime unhealthiness, reducing perceived attractiveness; whereas occluding the face by objects, other than face mask, may tap into a face-specific positivity bias. This latter interpretation is supported by a recent study by Orghian and Hidalgo (2020) who reported that photos of incomplete faces are perceived as more attractive, although we hasten to add that this study did not consider occlusion of the bottom half of the face, but rather manipulations to photos of faces such as blurring, cropping two-thirds of an entire face, and randomly removing pixel images.

The present study sought to extend these novel results with a closer inspection of the effect of a face mask on the perception of facial attractiveness, by comparing masked faces to when the bottom- or top-half of the faces are cropped. In doing so we can test whether the results found using a Japanese sample by Miyazaki and Kawahara (2016) extend to another culture; specifically, their finding that unlike occlusion which increased perceived attractiveness, only face masks reduce the perceived attractiveness of high (and average female) faces, but not low attractive faces. A similar outcome would suggest that the pattern reported by Miyazaki and Kawahara (2016), is a general phenomenon, and not a culturally-specific one. Failure to replicate their findings would exclude this possibility. For instance, in keeping with the positivity bias suggested by Orghian and Hidalgo (2020), we might find that masks increase the perception of attractiveness. An alternative possibility is that masks will interfere with the perception of features that are both attractive (e.g., symmetry) and unattractive (e.g., asymmetry) the result being that attractive faces when masked may be perceived as less attractive but unattractive faces when masked may appear more attractive.

## Study 1

In the first study, we tested the effect of a facial mask on perception of attractiveness using a sample of young people's faces with a neutral facial expression.

### Method

#### Participants

A total of 164 individuals (58 men and 106 women), aging between 18 and 75 years of age (*M* = 40.6, SD = 14.3), were recruited from TurkPrime panel as participants. A total of 92 participants (56.1%) reported being married, 1.2% reported widowed and an additional 8.5% reported being divorced or separated, while 24.4% reported being single, and 9.8% in a relationship. In terms of their highest educational degree, 30.5% had a high school diploma, 7.3% had a post-secondary diploma, 25.0% of the participants had an undergraduate degree, one reported elementary school and 36.6% had a post-graduate degree.

#### Stimuli and procedure

**Fig. 1:**
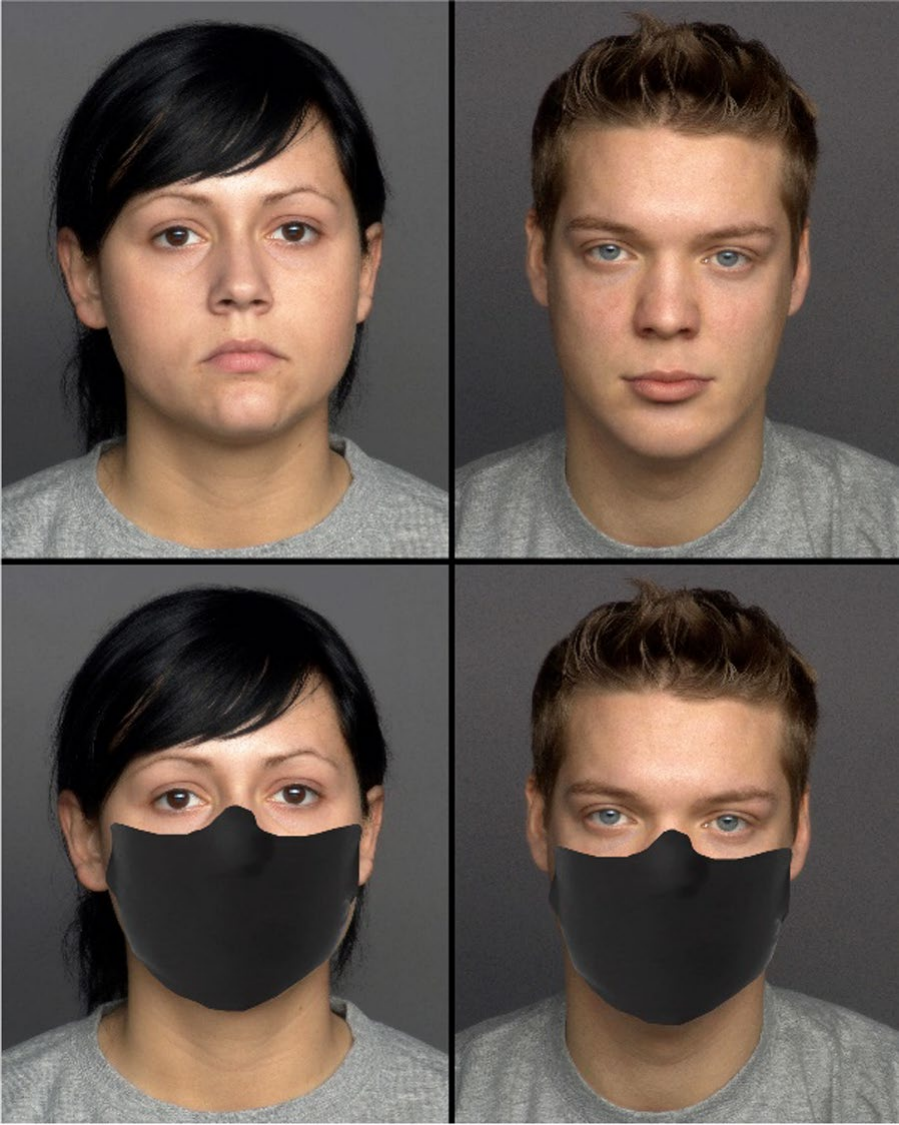
Examples of young female and male faces used in Study 1

Images of 25 male and 25 female faces, aged between 19 and 31 years and with a neutral expression, were obtained from the FACES database (Ebner et al., 2010), resulting in 50 stimuli. Another set of 50 stimuli of the same identities were created by superimposing a facial mask on the original images. Each set of stimuli (masked or unmasked) were randomised and presented in separate blocks. After consenting to participate in the study, participants answered sociodemographic questions. A within-subjects design was used, and participants randomly observed either the block with facial masks first or the block without masks first. Participants were asked to respond to the question “How attractive do you find this man?” or “How attractive do you find this woman?” on a 7-point scale, from 1 (not at all) to 7 (very) (Fig. 1).

#### Data analysis

The average ratings of attractiveness for the original unmasked stimuli was calculated and the stimuli were grouped as above (high attractiveness) and below (low attractiveness) the average. All post hoc comparisons throughout the results of this and next studies were performed using Bonferroni correction, and this is reflected in the *p* values. A G*Power analysis for a 2 × 2 × 2 × 2 mixed effects design indicated that 92 participants would be sufficient to detect a small effect size (*f* = 0.10, *β* = 0.80). In our studies we recruited participants almost twice the needed size (~ 180).

### Results

A linear mixed model was conducted to investigate the effect of presence/absence of facial mask, stimuli sex, participants’ sex, and attractiveness group (low and high) on the ratings of attractiveness in young people faces, with participants as a random factor. Results showed significant main effects for facial mask, stimuli sex, participant sex, and attractiveness group (see Additional file 1: Table S1 for details). The main effect of mask was further qualified by two 2-way interactions: Mask × Attractiveness Group, and Mask × Stimuli Sex.

**Fig. 2:**
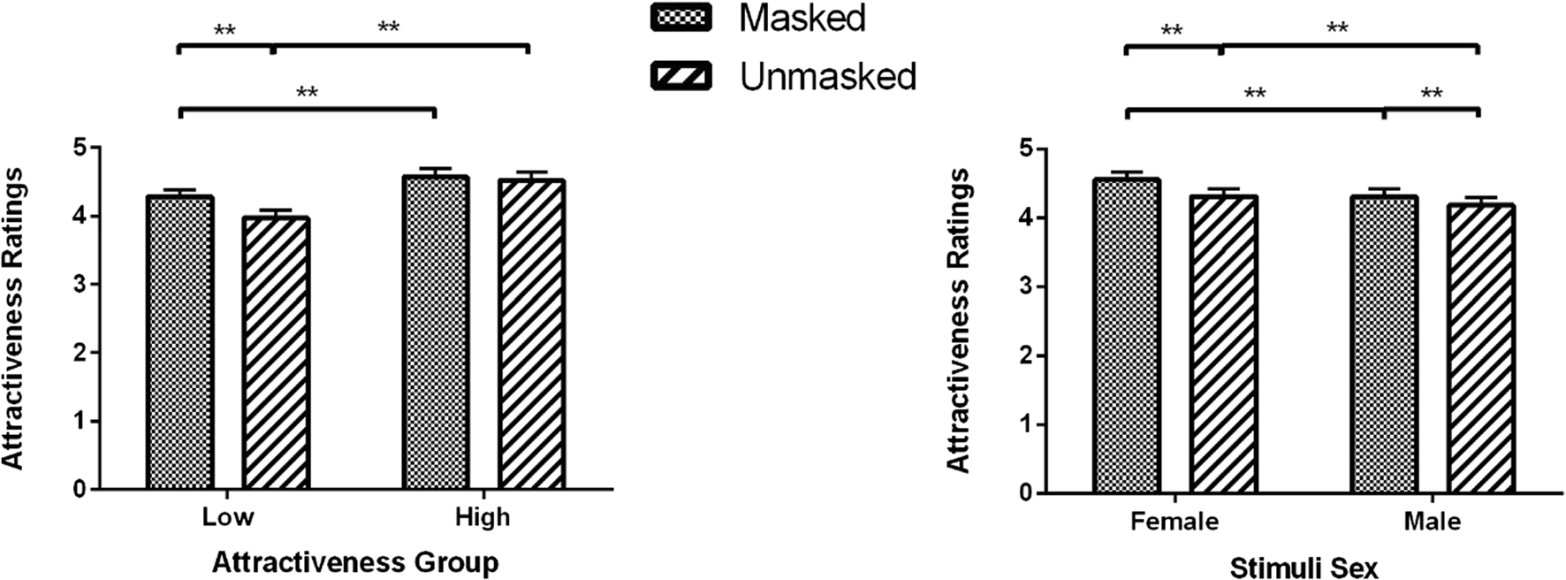
Attractiveness ratings (Means and SEM) for masked and unmasked young faces as a function of attractiveness group and stimuli sex. *p < 0.05, **p < 0.01

The Mask × Attractiveness Group interaction showed that for the low attractiveness group, participants rated faces with masks (*M* = 4.28, SEM = 0.11) as more attractive compared to their unmasked faces (*M* = 3.97, SEM = 0.11, *p* < 0.001); while in the high attractiveness group the ratings were not significantly different between masked (*M* = 4.57, SEM = 0.12) and unmasked of attractive faces (*M* = 4.52, SEM = 0.12, *p* = 0.999) (Fig. 2, left panel).

The Mask × Stimuli Sex interaction (as seen in Fig. 2, right panel) reflects that as females are more attractive than males overall (*p*s < 0.012) and masks increase the attractiveness of both females and males (*p*s < 0.001), a masked male (*M* = 4.30, SEM = 0.12) is perceived as attractive as an unmasked female (*M* = 4.31, SEM = 0.11, *p* = 0.999), but a masked female (*M* = 4.55, SEM = 0.11) is significantly more attractive than an unmasked male (*M* = 4.18, SEM = 0.12, *p* < 0.001).

### Discussion

Results of the first study showed that facial masks increased ratings of attractiveness for those faces that were less attractive than average, while having no effect on above average attractive faces. Such results are in contrast with those of Miyazaki and Kawahara (2016), who reported an overall negative effect of facial masks on ratings of attractiveness. However, our results converge partially with those of Orghian and Hidalgo (2020) who showed a positive bias for incomplete faces. Nonetheless, our result is limited to a sample of young faces and might not be generalized to a broader population (i.e., older adults).

Previous research has shown that individuals consider younger faces more attractive than older faces (Ebner, 2008; Kwart et al., 2012). It is suggested that age-related features in older faces such as lines, wrinkles, and furrows on skin results in their being perceived as relatively less attractive than younger faces (Berry & McArthur, 1986; Matts et al., 2007).

To the extent that our finding in Study 1 is robust, we would expect masks to increase the attractiveness only of older adult faces that are less attractive on average. Alternatively, because older adults are generally less attractive than younger faces, one might expect that masks will increase the attractiveness rating of all older faces. There is a second reason to expect this outcome. By covering the lower part of the face, wearing facial masks would reduce the evidence of age-related changes to the face, thereby making all of the older faces more attractive.

## Study 2

In the second study we aimed to extend the results found for young faces to old people faces and test whether the effect from Study 1 holds true when using face stimuli with a different age range.

### Method

#### Participants

A total of 181 individuals (72 men and 109 women), aging between 18 and 90 years of age (*M* = 45.3, SD = 16.9), were recruited from TurkPrime panel as participants. A total of 73 participants (40.3%) reported being married, 4.4% reported widowed and an additional 11.6% reported being divorced or separated, while 30.9% reported being single, and 12.7% in a relationship. In terms of their highest educational degree, 45.3% had a high school diploma, 11.6% had a post-secondary diploma, 24.9% of the participants had an undergraduate degree, one (0.6%) reported elementary school and 17.7% had a post-graduate degree.

#### Stimuli and procedure

**Fig. 3:**
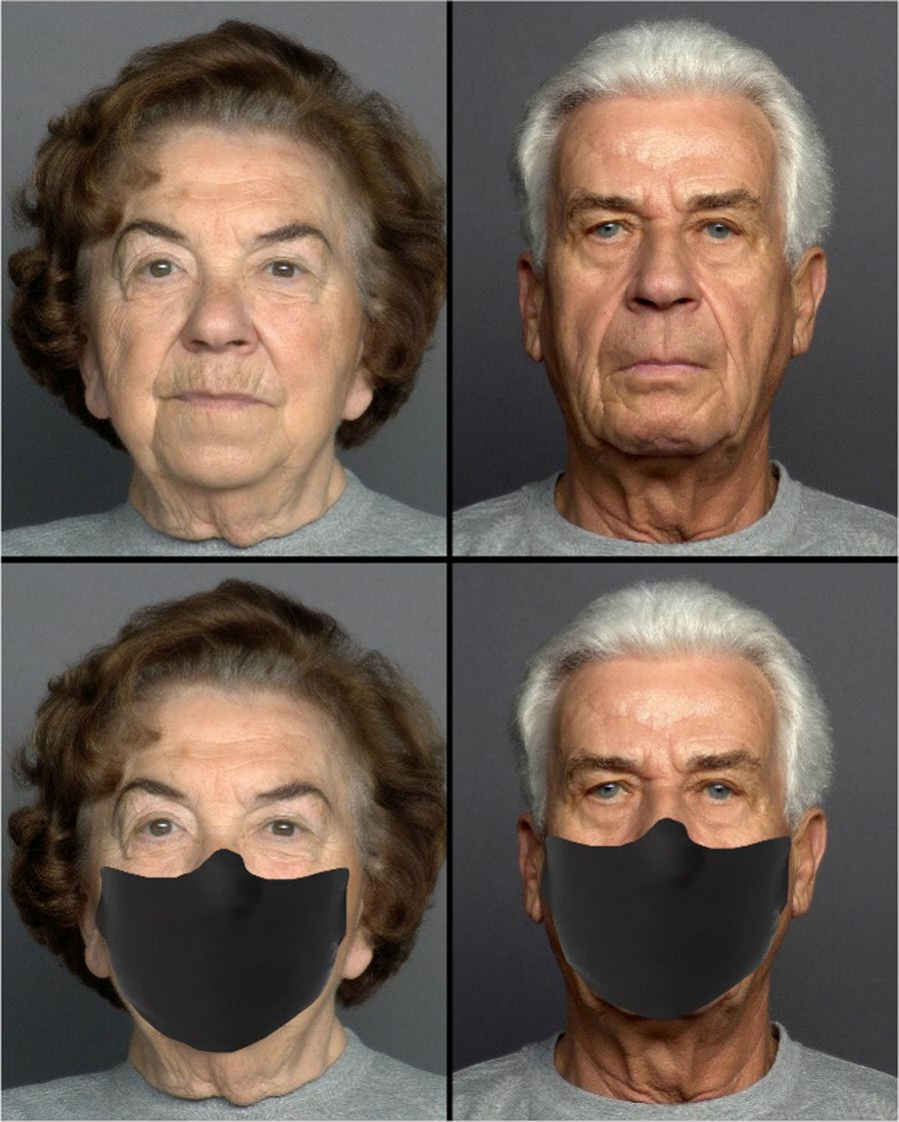
Examples of old male and female faces used in Study 2

Images of 25 male and 25 female faces, aged between 69 and 80 years, with neutral expression were obtained from FACES database (Ebner et al., 2010), resulting in 50 stimuli. Similar to Study 1, another set of 50 masked stimuli of the same identities were created. The procedure was the same as Study 1 (Fig. 3).

### Results

As in Study 1, a linear mixed model was conducted to investigate the effect of presence/absence of facial mask, stimuli sex, participants’ sex, and attractiveness group (low and high) on the ratings of attractiveness of old faces, with participants as a random factor. Results showed significant main effects for facial mask, stimuli sex, and attractiveness group (see Additional file 1: Table S2 for details). The mask main effect was further qualified by two 2-way interactions: Mask × Attractiveness Group, and Mask × Stimuli Sex.

**Fig. 4:**
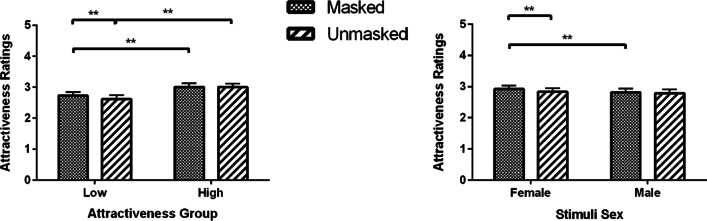
Attractiveness ratings (Means and SEM) for masked and unmasked old faces as a function of attractiveness group and stimuli sex. *p < 0.05, **p < 0.01

The Mask × Attractiveness Group interaction showed that for the low attractiveness group, participants rated faces with masks (*M* = 2.73, SEM = 0.12) as more attractive compared to unmasked faces (*M* = 2.62, SEM = 0.12, *p* < 0.001); while in the high attractiveness group the ratings were not significantly different for masked (*M* = 3.01, SEM = 0.12) and unmasked of attractive faces (*M* = 3.00, SEM = 0.12, *p* = 0.999) (Fig. 4; left panel).

Results for the Mask × Stimuli Sex interaction showed that participants rated masked female faces (*M* = 2.92, SEM = 0.12) as more attractive than unmasked female faces (*M* = 2.83, SEM = 0.12, *p* < 0.001), whereas masking male faces had no effect (Masked: *M* = 2.81, SEM = 0.12; Unmasked: *M* = 2.79, SEM = 0.12, *p* = 0.999; see Fig. 4, right panel).

### Discussion

Results of the second study using a sample of faces from older adults replicated the findings from the first study in which participants rated the faces of younger adults. Though the ratings of attractiveness were markedly lower for the old faces in Study 2 compared to the ratings of young faces in Study 1, and the overall effect of masks on males was nonsignificant, our core finding was the same. That is, masking faces that were less attractive than average increased their perceived attractiveness, but no comparable change was observed when above average faces were masked. Therefore, the effect of a mask on facial attractiveness extends across the ages and suggests a broader and more general phenomenon of face masks on perception of facial attractiveness. Moreover, in the first two experiments, we superimposed facial masks on faces, rather than creating stimuli by taking photos of the models both with and without masks. Although this may have resulted in poorer, loosely fitted masks, less realistic stimuli, and generally less appealing stimuli; despite these factors and speaking to the potential robustness of our results, we found that less attractive faces with masks are considered more attractive. To examine whether this effect is mask-specific or extends to the general situation when the information from the lower half of the face is unavailable, Study 3 cropped the area of the face that had been masked in Study 1.

## Study 3

The study examined whether the results found in Study 1 with young adults, and replicated with older adults, would also be observed when the lower half of the face was cropped rather than masked. We tested these using the faces in Study 1.

### Method

#### Participants

A total of 173 individuals (70 men and 103 women), aging between 18 and 90 years of age (*M* = 49.9, SD = 18.3), were recruited from TurkPrime panel as participants. A total of 84 participants (48.6%) reported being married, 6.9% reported widowed and an additional 9.8% reported being divorced or separated, while 26.0% reported being single, and 8.7% in a relationship. In terms of their highest educational degree, 39.9% had a high school diploma, 11.6% had a post-secondary diploma, 29.5% of the participants had an undergraduate degree, one (0.6%) reported elementary school and 18.5% had a post-graduate degree.

#### Stimuli and procedure

**Fig. 5:**
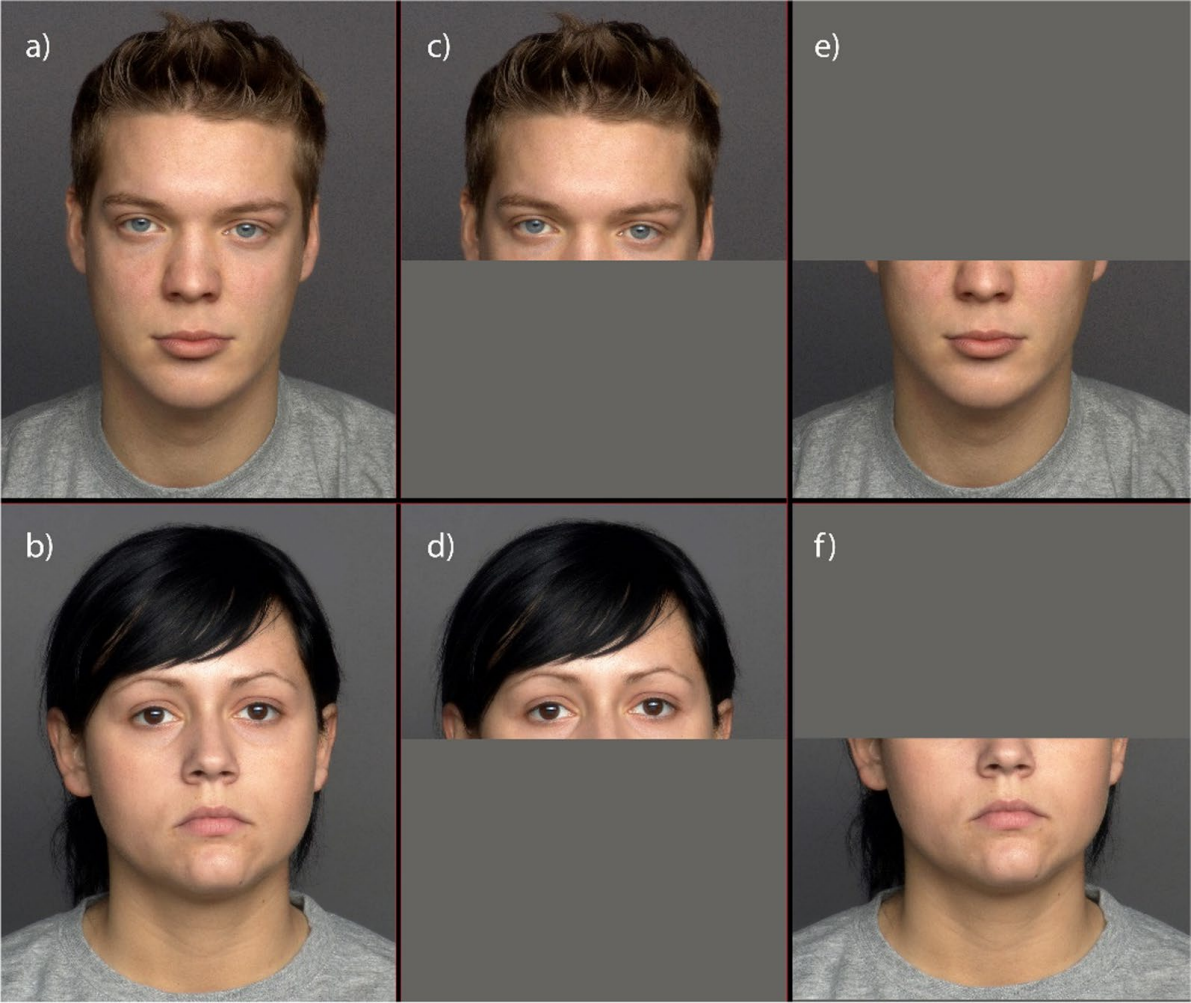
Examples of full (**a** and **b**), and upper half male and female faces (**c** and **d**) used in Study 3; examples of full (**a** and **b**) and lower half faces (**e** and **f**) used in Study 4

The 50 young unmasked faces used in Study 1 served as stimuli. Moreover, another set of 50 stimuli of the same identities were created by cropping the lower face of the stimuli (see Fig. 5). The procedure was the same as Study 1.

### Results

A linear mixed model was conducted to investigate the effect of face (half and full), stimuli sex, participants’ sex and attractiveness group (low and high) on the ratings of attractiveness, with participants as a random factor. Results returned significant main effects for face, stimuli sex, participant sex, and attractiveness group (see Additional file 1: Table S3 for details). The face main effect was further qualified by two 2-way interactions: Face × Attractiveness Group and Face × Stimuli Sex.

**Fig. 6:**
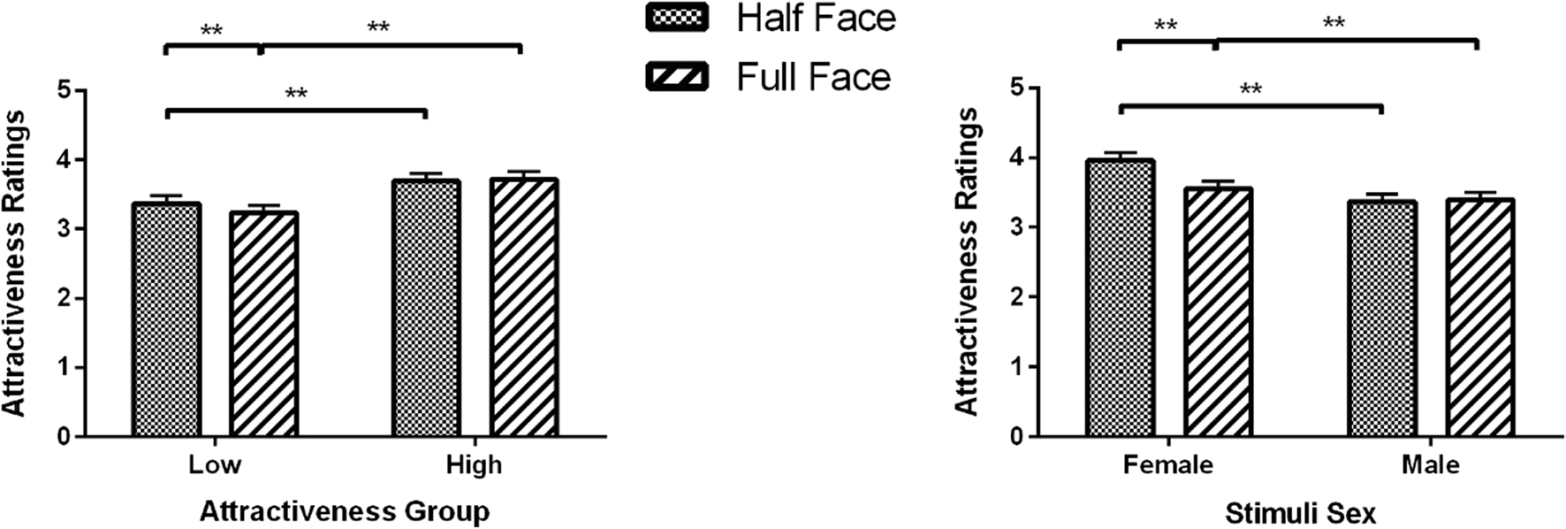
Attractiveness ratings (Means and SEM) for upper half and full faces as a function of attractiveness group and stimuli sex. *p < 0.05, **p < 0.01

The Face × Attractiveness Group interaction showed that for low attractiveness group, participants rated half faces (*M* = 3.37, SEM = 0.11) more attractive compared to full faces (*M* = 3.23, SEM = 0.11, *p* < 0.001); while the ratings were not significantly different for half (*M* = 3.69, SEM = 0.11) and full high attractive faces (*M* = 3.72, SEM = 0.11, *p* = 0.999) (Fig. 6; left panel).

Results for Face × Stimuli Sex interaction showed that participants rated female half faces (*M* = 3.69, SEM = 0.11) more attractive than female complete faces (*M* = 3.55, SEM = 0.11, *p* < 0.001). Male half (*M* = 3.37, SEM = 0.11) and full faces (*M* = 3.39, SEM = 0.11, *p* = 0.999) were not rated differently (Fig. 6; right panel).

### Discussion

The present results replicate the key pattern observed in the previous studies for masked and unmasked young and older adult faces. Removing the information from the lower half of the faces that are below average in attractiveness has the effect of increasing their perceived attractiveness. A comparable bump in perceived attractiveness is not observed for above average faces. These results suggest that the effect we have found is not mask-specific, again separating our results from those of Miyazaki and Kawahara (2016). Recall that Miyazaki and Kawahara (2016) had found that any effect of face masks was to reduce the perceived attractiveness of faces, and that this was unique to masks. When a notebook or a card occluded the lower half of a face, the effect was to enhance the perceived attractiveness of unattractive faces. On that score the present results are convergent with Miyazaki and Kawahara (2016). Similarly, in their card-occlusion experiment, which removed a potential confound in their notebook-occlusion experiment, Miyazaki and Kawahara (2016) found no effect of occlusion on attractive faces (i.e., low attractive faces were more attractive when occluded, but attractive faces were not), thereby replicating our present result and those of our previous two experiments. Collectively, these data suggest that the primary difference between the results of our study and those of Miyazaki and Kawahara (2016), may be attributed to the lack of association between illness and face masks in North America, which Miyazaki and Kawahara argued was driving their effect of face masks in Japan.

Our final study sought to determine if our findings are specific to removing the information from the lower half of the face. Or if it is also observed when the information from the upper half of the face is unavailable.

## Study 4

Our results showing that faces that are below average are considered more attractive when information from the lower half of the face is unavailable is consistent with a positive bias (e.g., Orghian & Hidalgo, 2020); although our finding that it does not extend to the more attractive faces is not. Recall however that Orghian and Hidalgo did not consider occlusion of the bottom half of the face, but rather manipulations to photos of faces such as blurring, cropping two-thirds of an entire face, and randomly removing pixel images. So the methodologies between the two studies are profoundly different, and that may play a significant role in the discrepant findings. That said, it is also worth noting that Orghian and Hidalgo (2020) did find a larger positivity bias for atypical (putatively less attractive) faces than typical (more attractive) faces, which is broadly speaking convergent with our findings. Thus, based on our study, and that of Orghian and Hidalgo, a reasonable prediction is that occluding the upper half of the face will increase the perceived attractiveness of less attractive faces than more attractive faces, to the extent that there is no effect at all on the highly attractive faces, as we found in Studies 1–3.

However, eye tracking studies have shown that the eyes are the most important region of the face when individuals judge facial attractiveness (Kwart et al., 2012; Nguyen et al., 2009). If the eye region is crucial for judgements of facial attractiveness then it is reasonable to expect that our results may be specific to the case where the eyes are visible. In other words, an alternative prediction is that cropping the upper half of a face in the present study may abolish the pattern of results we observed across three previous studies, which had involved masking or cropping the bottom half of the face.

### Method

#### Participants

A total of 180 individuals (68 men and 112 women), aging between 18 and 78 years of age (*M* = 48.6, SD = 16.8), were recruited from TurkPrime panel as participants. A total of 76 participants (42.2%) reported being married, 23.3% reported widowed, being divorced or separated, while 26.1% reported being single, and 8.3% in a relationship. In terms of their highest educational degree, 42.2% had a high school diploma, 11.1% had a post-secondary diploma, 23.3% of the participants had an undergraduate degree, 1.1% reported elementary school and 22.2% had a post-graduate degree.

#### Stimuli and procedure

The 50 faces used in Study 1 were employed as stimuli. Another set of 50 stimuli of the same identities were created by cropping the upper face of the stimuli (see Fig. 5). The procedure was the same as Study 1.

### Results

A linear mixed model was conducted to investigate the effect of face (half and full), stimuli sex, participants’ sex and attractiveness group (low and high) on the ratings of attractiveness, with participants as a random factor. Results showed significant main effects for face, stimuli sex, and attractiveness group (see Additional file 1: Table S4 for details). The face main effect was qualified by two 2-way interactions: Face × Attractiveness Group, and Face × Stimuli Sex.

**Fig. 7:**
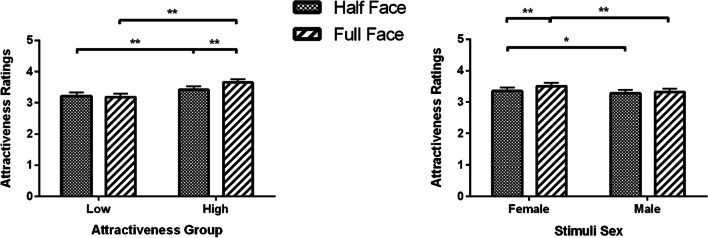
Attractiveness ratings (Means and SEM) for upper half and full faces as a function of attractiveness group and stimuli sex. *p < 0.05, **p < 0.01

Results for Face × Attractiveness Group interaction showed that for the high attractiveness group, participants rated full faces (*M* = 3.65, SEM = 0.11) as more attractive compared to half faces (*M* = 3.42, SEM = 0.11, *p* < 0.001); while the ratings were not significantly different between full faces (*M* = 3.18, SEM = 0.11, *p* = 0.600) and half faces (*M* = 3.22, SEM = 0.11) in the low attractiveness group (Fig. 7; left panel).

Results for Face × Stimuli Sex interaction showed that participants rated female full faces (*M* = 3.50, SEM = 0.11) more attractive than female half faces (*M* = 3.35, SEM = 0.11, *p* < 0.001). Male full faces (*M* = 3.32, SEM = 0.11) and half faces (*M* = 3.28, SEM = 0.11) were not rated differently (*p* = 0.420; see Fig. 7, right panel).

### Discussion

Results of the fourth study, comparing the attractiveness ratings of lower faces with complete faces showed that for the high attractiveness group, participants rated full faces as more attractive than their lower half faces. For the low attractiveness group there was no significant difference between full and half faces. This finding is precisely the opposite to what we found in Studies 1–3, where the low attractiveness group was rated as more attractive when only the upper half of their face was visible; and no effect of masking or cropping was found for the high attractiveness group. Thus, our present study indicates that a positivity bias for incomplete faces does not hold (Orghian & Hidalgo, 2020), at least in the rather unique situation where the entire upper half of the face is removed. When this occurs, the perceived attractiveness of half faces is the same as full faces when the full faces are unattractive, or less than full faces when the full faces are highly attractive.

The present findings do, however, support the argument that the eye region is an important area in individuals’ judgement of facial attractiveness (Kwart et al., 2012; Nguyen et al., 2009), and as a result its impact is felt most strongly by those who are most attractive. This also helps to explain why masking or cropping the lower half of the face in our previous studies had a nominal effect on those who were above average in attractiveness, i.e., their vital and highly attractive eye region was preserved.

## General discussion

In the current research we investigated the effect of facial masks on the perception of facial attraction. Specifically, we tested whether below- and above-average full faces are equally affected by wearing facial masks. Our first study revealed that for younger faces a facial mask will increase the perceived attractiveness if the unmasked face is relatively unattractive, but wearing a mask will not help or hinder highly attractive faces. Study 2 revealed that this pattern of results generalises to old faces. And our Study 3 showed that the same effect occurs even when the bottom half of young faces are cropped rather than masked, i.e., the effect is not mask-specific.

Collectively, our first two studies, which show that face masks will help and never hinder one's perceived attractiveness conflicts with the findings of Miyazaki and Kawahara (2016) who showed that facial mask decrease the perceived attractiveness in a pre-COVID-19 pandemic era. Interestingly, the same lab has tested the same question recently during the pandemic and reported a similar result to our first two studies—attractiveness of below-average faces increased (Kamatani et al., 2021). A similar pattern of improvement in attractiveness ratings of unattractive faces is confirmed in another post-pandemic study (Patel et al., 2020), signifying the change in attitudes in response to social norms associated with mask wearing (Carbon, 2021). However, the results of Kamatani et al. (2021) for masked faces of above-average attractiveness showed a reduction in perception of attractiveness compared to unmasked faces—a result not found in the studies here and those of Patel et al. (2020). Such discrepancy might be a result of cultural differences (Japanese vs. Western) or due to differences in the stimuli used, in terms of their range of attractiveness.

Our final Study 4 examined if our previous findings are specific to the removal of information from the lower half of the face, or does it reflect a more general positivity bias where any incomplete face information is inferred to be attractive (Orghian & Hidalgo, 2020). The results did not support this possibility. When observers were asked to judge the attractiveness of faces that had the upper half of the face removed (including the eyes), the effect was to reduce the perceived attractiveness of highly attractive faces, and to have no positive effect on less attractive faces. This latter study demonstrates that in North America the effect of perceiving an incomplete face can be detrimental; and it supports previous work indicating that the eye-region has a special status in perceptions of facial attractiveness (Kwart et al., 2012; Nguyen et al., 2009). Indeed, the fact that in Studies 1–3 the eye region of the face was preserved helps to explain why masking or cropping the lower half of the face had no effect on the perceived attractiveness of highly attractive faces. Collectively, across four experiments, our study reveals that facial masks increase the perceived attractiveness of less attractive faces, while they do not affect those that are highly attractive. This finding applies to young and old faces, and it extends to other methods of isolating the region of the upper face; but it does not apply to the situation when the lower half of the face is isolated. These findings suggest that a positivity-bias enhances the perception of unattractive faces when only the upper face is visible, a finding that may not extend to attractive faces because of the perceptual weight placed on their eye-region.

## Supplementary Information

**Additional file 1.** **Table S1.** Estimates for the Effects of Facial Mask, Stimuli Sex, Participant Sex and Stimuli Attractiveness (Low vs. High) on the Ratings of Attractiveness in Young Faces. **Table S2.** Estimates for the Effects of Facial Mask, Stimuli Sex, Participant Sex and Stimuli Attractiveness (Low vs. High) on the Ratings of Attractiveness in Old Faces. **Table S3.** Estimates for the Effects of Face (Half vs. Full), Stimuli Sex, Participant Sex and Stimuli Attractiveness (Low vs. High) on the Ratings of Attractiveness. **Table S4.** Estimates for the Effects of Face (Lower Half vs. Full), Stimuli Sex, Participant Sex and Stimuli Attractiveness (Low vs. High) on the Ratings of Attractiveness.

## Data Availability

The datasets used and/or analysed during the current study are available from the corresponding author on reasonable request.
