# Supplementary material for: Unattractive faces are more attractive when the bottom-half is masked, an effect that reverses when the top-half is concealed
Source: Cogn Res Princ Implic. 2022 Jan 24;7:6. doi: 10.1186/s41235-022-00359-9 (PMC8785149; doi:10.1186/s41235-022-00359-9)
Supplement: Supplementary file 1 — Additional file 1. Table S1. Estimates for the Effects of Facial Mask, Stimuli Sex, Participant Sex and Stimuli Attractiveness (Low vs. High) on the Ratings of Attractiveness in Young Faces. Table S2. Estimates for the Effects of Facial Mask, Stimuli Sex, Participant Sex and Stimuli Attractiveness (Low vs. High) on the Ratings of Attractiveness in Old Faces. Table S3. Estimates for the Effects of Face (Half vs. Full), Stimuli Sex, Participant Sex and Stimuli Attractiveness (Low vs. High) on the Ratings of Attractiveness. Table S4. Estimates for the Effects of Face (Lower Half vs. Full), Stimuli Sex, Participant Sex and Stimuli Attractiveness (Low vs. High) on the Ratings of Attractiveness. [file 41235_2022_359_MOESM1_ESM.docx]

| Table S1. Estimates for the Effects of Facial Mask, Stimuli Sex, Participant Sex and Stimuli Attractiveness (Low vs. High) on the Ratings of Attractiveness in Young Faces | | | | | | | |
| --- | --- | --- | --- | --- | --- | --- | --- |
|  | | | 95% CI | |  | | |
| Effect | β | *SE* | Lower | Upper | *df* | *t* | *p* |
| (Intercept) | 4.33 | 0.11 | 4.11 | 4.56 | 163.48 | 38.54 | < .001*** |
| Mask (unmasked) | -0.18 | 0.02 | -0.23 | -0.13 | 16222 | -7.62 | < .001*** |
| Stimuli Sex (male) | -0.19 | 0.02 | -0.23 | -0.14 | 16222 | -7.93 | < .001*** |
| Participant Sex (male) | 0.67 | 0.22 | 0.23 | 1.12 | 163.48 | 3.00 | .003** |
| Attractiveness Group (low) | -0.42 | 0.02 | -0.47 | -0.38 | 16222 | -17.98 | < .001*** |
| Mask × Stimuli Sex | 0.12 | 0.05 | 0.02 | 0.21 | 16222 | 2.48 | .013* |
| Mask × Participant Sex | 0.12 | 0.05 | 0.03 | 0.22 | 16222 | 2.62 | .009** |
| Stimuli Sex × Participant Sex | -0.23 | 0.05 | -0.32 | -0.13 | 16222 | -4.81 | < .001*** |
| Mask × Attractiveness Group | -0.26 | 0.05 | -0.35 | -0.17 | 16222 | -5.48 | < .001*** |
| Stimuli Sex × Attractiveness Group | 0.02 | 0.05 | -0.07 | 0.11 | 16222 | 0.46 | 0.642 |
| Participant Sex × Attractiveness Group | 0.21 | 0.05 | 0.11 | 0.30 | 16222 | 4.34 | < .001*** |
| Mask × Stimuli Sex × Participant Sex | 0.09 | 0.09 | -0.10 | 0.27 | 16222 | 0.91 | 0.365 |
| Mask × Stimuli Sex × Attractiveness Group | 0.01 | 0.09 | -0.18 | 0.19 | 16222 | 0.01 | 0.992 |
| Mask × Participant Sex × Attractiveness Group | 0.04 | 0.09 | -0.14 | 0.23 | 16222 | 0.47 | 0.638 |
| Stimuli Sex × Participant Sex × Attractiveness Group | 0.04 | 0.09 | -0.15 | 0.22 | 16222 | 0.38 | 0.702 |
| Mask × Stimuli Sex × Participant Sex × Attractiveness Group | -0.19 | 0.19 | -0.56 | 0.18 | 16222 | -1.00 | 0.316 |
| Note. * *p* < .05, ** *p* < .01, *** *p* < .001 | | | | | | | |

| Table S2. Estimates for the Effects of Facial Mask, Stimuli Sex, Participant Sex and Stimuli Attractiveness (Low vs. High) on the Ratings of Attractiveness in Old Faces | | | | | | | |
| --- | --- | --- | --- | --- | --- | --- | --- |
|  | | | 95% CI | |  | | |
| Effect | β | *SE* | Lower | Upper | *Df* | *t* | *p* |
| (Intercept) | 2.84 | 0.11 | 2.62 | 3.06 | 179.04 | 24.78 | < .001*** |
| Mask (unmasked) | -0.06 | 0.01 | -0.08 | -0.03 | 17905 | -4.40 | < .001*** |
| Stimuli Sex (female) | 0.07 | 0.01 | 0.05 | 0.10 | 17905 | 5.76 | < .001*** |
| Participant Sex (male) | 0.19 | 0.23 | -0.25 | 0.64 | 179.04 | 0.85 | .398 |
| Attractiveness Group (low) | -0.33 | 0.01 | -0.36 | -0.30 | 17905 | -25.56 | < .001*** |
| Mask × Stimuli Sex | -0.07 | 0.03 | -0.12 | -0.02 | 17905 | -2.70 | .007** |
| Mask × Participant Sex | 0.14 | 0.03 | 0.09 | 0.19 | 17905 | 5.41 | < .001*** |
| Stimuli Sex × Participant Sex | -0.11 | 0.03 | -0.16 | -0.06 | 17905 | -4.41 | < .001*** |
| Mask × Attractiveness Group | -0.10 | 0.03 | -0.15 | -0.05 | 17905 | -3.95 | < .001*** |
| Stimuli Sex × Attractiveness Group | -0.10 | 0.03 | -0.15 | -0.05 | 17905 | -3.99 | < .001*** |
| Participant Sex × Attractiveness Group | 0.13 | 0.03 | 0.08 | 0.18 | 17905 | 5.03 | < .001*** |
| Mask × Stimuli Sex × Participant Sex | -0.01 | 0.05 | -0.11 | 0.09 | 17905 | -0.22 | .830 |
| Mask × Stimuli Sex × Attractiveness Group | -0.02 | 0.05 | -0.12 | 0.09 | 17905 | -0.29 | .769 |
| Mask × Participant Sex × Attractiveness Group | -0.02 | 0.05 | -0.12 | 0.08 | 17905 | -0.41 | .679 |
| Stimuli Sex × Participant Sex × Attractiveness Group | 0.14 | 0.05 | 0.04 | 0.24 | 17905 | 2.76 | .006** |
| Mask × Stimuli Sex × Participant Sex × Attractiveness Group | -0.02 | 0.10 | -0.23 | 0.18 | 17905 | -0.22 | .824 |
| Note. * *p* < .05, ** *p* < .01, *** *p* < .001 | | | | | | | |

| Table S3. Estimates for the Effects of Face (Half vs. Full), Stimuli Sex, Participant Sex and Stimuli Attractiveness (Low vs. High) on the Ratings of Attractiveness | | | | | | | |
| --- | --- | --- | --- | --- | --- | --- | --- |
|  | | | 95% CI | |  | | |
| Effect | β | *SE* | Lower | Upper | *df* | *t* | *p* |
| (Intercept) | 3.50 | 0.11 | 3.29 | 3.71 | 171.3 | 32.66 | < .001*** |
| Face (full) | -0.06 | 0.02 | -0.09 | -0.03 | 17113 | -3.56 | < .001*** |
| Stimuli Sex (female) | 0.24 | 0.02 | 0.20 | 0.27 | 17113 | 14.69 | < .001*** |
| Participant Sex (male) | 0.49 | 0.21 | 0.07 | 0.91 | 171.3 | 2.29 | 0.023* |
| Attractiveness Group (low) | -0.40 | 0.02 | -0.43 | -0.37 | 17113 | -25.02 | < .001*** |
| Face × Stimuli Sex | -0.15 | 0.03 | -0.22 | -0.09 | 17113 | -4.73 | < .001*** |
| Face × Participant Sex | 0.15 | 0.03 | 0.09 | 0.21 | 17113 | 4.71 | < .001*** |
| Stimuli Sex × Participant Sex | 0.17 | 0.03 | 0.10 | 0.23 | 17113 | 5.20 | < .001*** |
| Face × Attractiveness Group | -0.16 | 0.03 | -0.23 | -0.10 | 17113 | -5.11 | < .001*** |
| Stimuli Sex × Attractiveness Group | -0.20 | 0.03 | -0.26 | -0.13 | 17113 | -6.14 | < .001*** |
| Participant Sex × Attractiveness Group | 0.06 | 0.03 | -0.01 | 0.12 | 17113 | 1.72 | 0.085 |
| Face × Stimuli Sex × Participant Sex | 0.07 | 0.06 | -0.05 | 0.20 | 17113 | 1.15 | 0.249 |
| Face × Stimuli Sex × Attractiveness Group | -0.11 | 0.06 | -0.24 | 0.02 | 17113 | -1.71 | 0.086 |
| Face × Participant Sex × Attractiveness Group | -0.04 | 0.06 | -0.16 | 0.09 | 17113 | -0.55 | 0.586 |
| Stimuli Sex × Participant Sex × Attractiveness Group | -0.09 | 0.06 | -0.21 | 0.04 | 17113 | -1.33 | 0.184 |
| Face × Stimuli Sex × Participant Sex × Attractiveness Group | -0.01 | 0.13 | -0.26 | 0.25 | 17113 | -0.05 | 0.956 |
| Note. * *p* < .05, ** *p* < .01, *** *p* < .001 | | | | | | | |

| Table S4. Estimates for the Effects of Face (Lower Half vs. Full), Stimuli Sex, Participant Sex and Stimuli Attractiveness (Low vs. High) on the Ratings of Attractiveness | | | | | | | |
| --- | --- | --- | --- | --- | --- | --- | --- |
|  | | | 95% CI | |  | | |
| Effect | β | *SE* | Lower | Upper | *df* | *t* | *p* |
| (Intercept) | 3.36 | 0.11 | 3.15 | 3.58 | 178.1 | 30.39 | < .001*** |
| Face (full) | 0.10 | 0.02 | 0.06 | 0.13 | 17806 | 5.87 | < .001*** |
| Stimuli Sex (female) | 0.12 | 0.02 | 0.09 | 0.16 | 17806 | 7.58 | < .001*** |
| Participant Sex (male) | 0.24 | 0.22 | -0.19 | 0.67 | 178.1 | 1.08 | 0.280 |
| Attractiveness Group (low) | -0.33 | 0.02 | -0.37 | -0.30 | 17806 | -20.49 | < .001*** |
| Face × Stimuli Sex | 0.11 | 0.03 | 0.05 | 0.17 | 17806 | 3.37 | < .001*** |
| Face × Participant Sex | 0.02 | 0.03 | -0.04 | 0.09 | 17806 | 0.67 | 0.503 |
| Stimuli Sex × Participant Sex | 0.12 | 0.03 | 0.06 | 0.19 | 17806 | 3.82 | < .001*** |
| Face × Attractiveness Group | -0.27 | 0.03 | -0.33 | -0.20 | 17806 | -8.22 | < .001*** |
| Stimuli Sex × Attractiveness Group | -0.23 | 0.03 | -0.29 | -0.16 | 17806 | -6.94 | < .001*** |
| Participant Sex × Attractiveness Group | -0.04 | 0.03 | -0.10 | 0.03 | 17806 | -1.16 | 0.246 |
| Face × Stimuli Sex × Participant Sex | -0.09 | 0.07 | -0.22 | 0.04 | 17806 | -1.37 | 0.170 |
| Face × Stimuli Sex × Attractiveness Group | -0.08 | 0.07 | -0.21 | 0.04 | 17806 | -1.27 | 0.203 |
| Face × Participant Sex × Attractiveness Group | 0.00 | 0.07 | -0.13 | 0.12 | 17806 | -0.07 | 0.947 |
| Stimuli Sex × Participant Sex × Attractiveness Group | -0.14 | 0.07 | -0.27 | -0.02 | 17806 | -2.22 | 0.026* |
| Face × Stimuli Sex × Participant Sex × Attractiveness Group | -0.12 | 0.13 | -0.37 | 0.14 | 17806 | -0.89 | 0.374 |
| Note. * *p* < .05, ** *p* < .01, *** *p* < .001 | | | | | | | |
